# Effect of magnetic MMP removal on long‐term dentin collagen stability

**DOI:** 10.1111/eos.70053

**Published:** 2025-11-19

**Authors:** Walter Zenobi, Salvatore Sauro, Davino Machado Andrade Neto, Karen Evellin Moura Cordeiro, Francisco Avelino, Diego Lomonaco, Pierre Basilio Almeida Fechine, Yu Fu Chou, Garrit Koller, Thiago Soares Porto, Victor Pinheiro Feitosa

**Affiliations:** ^1^ Postgraduate Program of Dentistry Federal University of Ceara Fortaleza Brazil; ^2^ Dental Biomaterials & Minimally Invasive Dentistry, Department of Dentistry University CEU Cardenal Herrera Valencia Spain; ^3^ Department of Materials and Metallurgic Engineering Federal University of Ceara Fortaleza Brazil; ^4^ Department of Organic and Inorganic Chemistry Federal University of Ceara Fortaleza Brazil; ^5^ Department of Analytic and Physics‐Chemistry Federal University of Ceara Fortaleza Brazil; ^6^ King's College London Dental Institute London UK; ^7^ Department of Operative Dentistry, College of Dentistry University of Iowa Iowa City Iowa USA

**Keywords:** adhesion, dentin, durability, Fe_3_O_4_ nanoparticles, metalloproteinase

## Abstract

Several matrix metalloproteinase (MMP) inhibitors have been investigated for their ability to improve dentin‐bond longevity. However, MMPs tend to reactivate over time, especially using simplified etch‐and‐rinse adhesives. This study investigated a novel magnetic removal of dentinal MMPs on bonding durability, MMP inhibition and collagen degradation. Magnetic nanoparticles (Fe_3_O_4_) were synthesized, characterized, and incorporated in water‐based gels at 2 wt% (MAG‐2) or 20 wt% (MAG‐20). A placebo and 2 wt% chlorhexidine (CHX) digluconate gel were employed as control treatments. Human teeth were acid‐etched, pretreated with gels (external magnetic motion was created in the MAG groups), and bonded to composite using a simplified adhesive. Specimens were subjected to microtensile bond strength test after 24 h or 1 year of water storage. Interface release of hydroxyproline was assessed. Presence of MMPs was analyzed by confocal microscopy in situ zymography. Data were statistically analyzed using two‐way ANOVA and Tukey's test. MAG‐2 was the only treatment providing stable bond strength, revealing lower MMP activity than CHX. MAG‐2 produced an evident removal of MMPs compared to CHX, MAG‐20, and placebo. The innovative magnetic treatment of dentin was able to effectively remove MMPs when used at a 2 wt% concentration, arresting dentin collagen degradation at the dentin‐bonded and achieving stable bonding over time.

## INTRODUCTION

Physicochemical and bonding properties of dental adhesives have been improved by numerous investigations focused on the chemical balance between their hydrophilic and hydrophobic components [1, 2]. However, the ultimate solution for longevity when bonding composites onto dentin has not yet been achieved. Regardless of the application technique, dental adhesives lose their dentin bonding performance over time, and there is consensus in the literature that loss of bond strength is related to the degradation of the hybrid layer [3].

Indeed, upon adhesive application to acid‐etched dentin, resin‐sparse collagen fibrils are detected within the hybrid layer, thresholding a suitable area for initial degradation [4]. Proteases such as matrix metalloproteinases (MMPs) are secreted by odontoblasts during dentinogenesis, and remain physiologically inactive in mineralized tissues [5]. After dentin demineralization due to etch‐and‐rinse bonding procedures or biological processes (e.g., caries and erosion), several MMPs are activated [6]. These enzymes are responsible for major collagen degradation within the hybrid layer and initial bond strength reduction of composite restorations [7, 8].

Several MMP inhibitors have been proposed to preserve unprotected collagen fibrils and reduce the degradation of the hybrid layer. For instance, even at low concentrations, chlorhexidine (CHX) showed a striking ability to inhibit MMPs 2, 8, and 9 [7]. Nevertheless, treatment with MMP‐inhibitors is reversible [9], since they only slow down the degradation process. To our knowledge, no investigations have attempted to extract these enzymes from the dentin organic matrix in situ, hampering our understanding of collagen degradation within the dentin‐bonded interface.

Thus, the aim of this study was to develop an innovative treatment based on magnetic removal of the metalloproteinases responsible for enzyme‐mediated collagen degradation within the hybrid layers, providing extended durability of resin–dentin bonds. This study tested the hypotheses that (i) such an innovative treatment would remove MMPs from the dentin matrix and (ii) improve the longevity of the bond strength to dentin.

## MATERIAL AND METHODS

### Synthesis of branched‐polyethylenimine‐coated Fe_3_O_4_ nanoparticles

**FIGURE 1:**
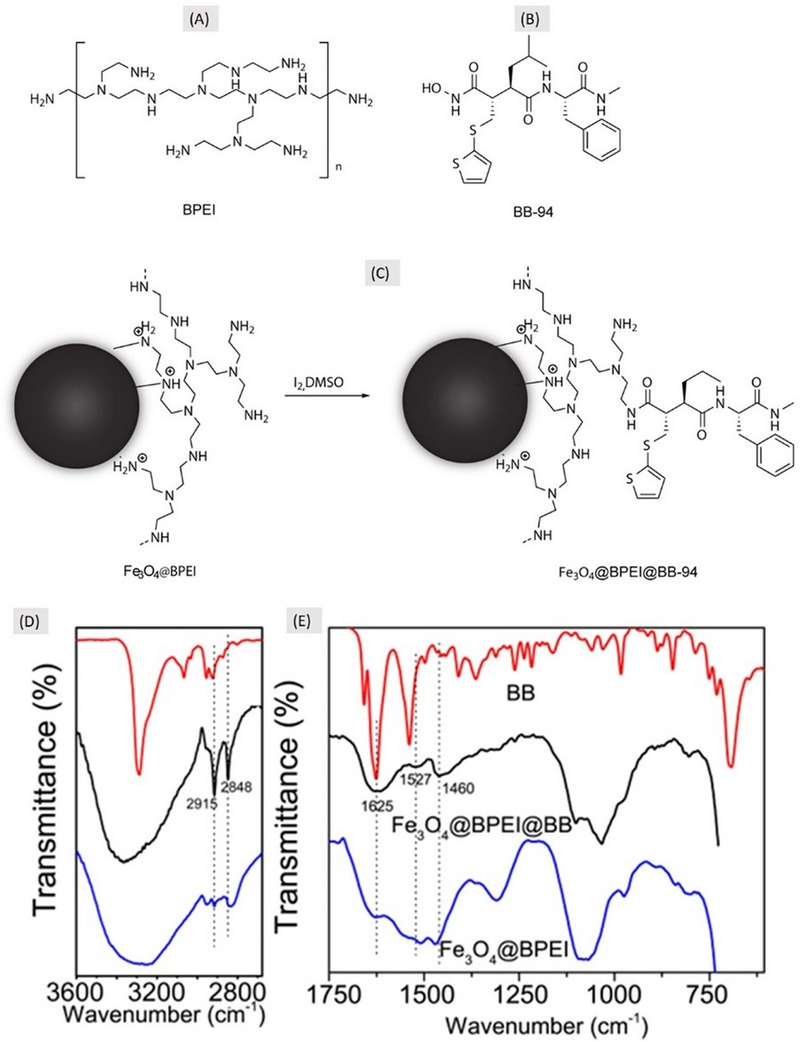
Scheme of the synthesis of nanoparticles for metalloproteinase magnetic removal. Chemical structure of BPEI and BB94 are depicted in (A) and (B), respectively. (C) Chemical strategy to anchor BB94 on the surface of presynthesized Fe_3_O_4_@BPEI nanoparticles to produce the final product for magnetic removal of matrix metalloproteinase (MMPs). (D) Fourier‐transform infrared spectroscopy (FTIR) spectra of the BB94 (batimastat), Fe_3_O_4_@BPEI@BB and Fe_3_O_4_@BPEI separated in the two main ranges to confirm the synthesis. (D) 2860–3600 cm^‒1^ range and (E) 615–1750 cm^‒1^ range. Dotted lines indicate peaks of BB94 presented/modified in magnetic nanoparticles with batimastat (Fe_3_O_4_@BPEI@BB), the final product (middle spectrum).

Fe_3_O_4_ nanoparticles [10] were functionalized by amine groups through branched‐polyethylenimine (BPEI) coating (Figure 1). The coating process was performed using the sonochemistry approach. Briefly, 1.16 g of FeSO_4_·7H_2_O and 1.85 g of FeCl_3_·6H_2_O were dissolved in 15 mL of deionized water and heated at 60°C. Then, this iron solution was sonicated for 60s using an ultrasonic probe. Afterward, 7 mL of concentrated NH_4_OH solution were added to the reaction medium, which remained under sonication for 4 min. Finally, 4 mL of aqueous solution containing 1 g of BPEI were added to reaction and left under sonication for an additional 4 min.

To remove excess NH_4_OH and unbounded BPEI, the resultant nanoparticles were washed seven times with acetone [10]. The nanoparticles were dispersed in deionized water and centrifuged for 10 min at 3000 rpm to remove nanoclusters. The remaining specimen was labeled as Fe_3_O_4_@BPEI. The amount of Fe_3_O_4_@BPEI in the aqueous suspension was calculated through gravimetry. All reagents were purchased from Sigma‐Aldrich.

### Anchoring batimastat (BB94) to Fe_3_O_4_@BPEI

The coupling reaction of BB94 (batimastat, Figure 1) on the magnetic nanoparticles was performed by an iodine‐mediated oxidation reaction, following the general strategy described by Krishnamurthy et al [11]. The anchoring occurred through the hydroxamic functionality of BB94 and the terminal amine groups of Fe_3_O_4_@BPEI, via the formation of acyl‐nitroso intermediates that predominantly react under mild conditions to yield amide bonds, as shown in Figure 1C.

An aqueous suspension containing 6 mg of Fe_3_O_4_@BPEI was magnetically separated and re‐suspended in 2 mL of dimethyl sulfoxide (DMSO). Subsequently, 2.0 mg of BB94 and 2.7 mg of iodine (I_2_) were solubilized in 3 mL of DMSO and added to the Fe_3_O_4_@BPEI suspension. The reaction medium remained under stirring in room temperature for 1 h. At the end of reaction, BB94‐modified nanoparticles were magnetic separated and washed four times with 5 mL of methanol to ensure complete removal of DMSO. Finally, the samples were dried under vacuum and labeled as Fe_3_O_4_@BPEI@BB94, which represents the final nanoparticles for magnetic removal of metalloproteinases (NMR‐MMPs).

### Fourier‐transform infrared spectroscopy

Samples of all products were characterized by Fourier‐transform infrared spectroscopy (FTIR) spectrophotometry (Spectrum Frontier, Perkin‐Elmer) equipped with attenuated total reflectance crystal (ATR‐FTIR). A spectral range of 4000–550 cm^−1^ with 4 cm^−1^ resolution was used. FTIR spectra were obtained in triplicate for each product, and processed for baseline correction and normalization.

### Preparation of dentin specimens

Twenty‐four extracted sound human third molars were selected after IRB approval (CAAE: 40416020.5.0000.9267) of Federal University of Ceara. They were stored, cut to expose flat dentin and a smear‐layer was created as described in a previous study [12]. Specimens were randomly divided in four treatment groups (*n* = 6/group): Control, treated with a placebo gel, 2% CHX, treated with a CHX gel; MAG‐2, treated with a gel with 2% NMR‐MMP; and MAG‐20, treated with a gel with 20% NMR‐MMP. Based on previous investigations, we calculated that six specimens per group would suffice to obtain minimal 80% power at 5% significance level for the detection of 20% difference in mean in microtensile bond strength and 40% difference in the mean hydroxyproline outcome.

All specimens were etched using a 37% phosphoric acid gel for 15 s. In the control group, a placebo gel containing distilled water without addition of MMP inhibitors or nanoparticles was applied onto the dentin for 60 s followed by water‐rinsing for 30 s. Specimens in CHX group were treated with a gel (2 wt%) of CHX digluconate (Sigma‐Aldrich) and used as positive control. In the group MAG‐2, the acid‐etched specimens were treated with a gel containing 2 wt% of NMR‐MMPs. In group MAG‐20 the specimens were treatment with a gel containing same nanoparticles (NMR‐MMPs) at 20 wt%. All gels were prepared with 30 wt% aerosil silica as thickener, and they were applied as described for the control followed by vigorous rinsing with water prior to adhesive application. However, before rising in the NMR‐MMPs groups, a neodymium magnet with 1.4T maximum internal field, 0.54T surface field (K&J Magnetics) was kept for 60 s at a 3 mm distance from the dentin to remove MMPs attached to magnetic nanoparticles. Two‐step etch‐and‐rinse adhesive Prime&Bond 2.1 (Dentsply) was applied according to the manufacturers' recommendations in two coats with final 20 s light‐curing. Afterward, a TPH Spectrum composite (Dentsply) build‐up was created incrementally. Light‐curing was performed with Valo LED unit (Ultradent) with 1200 mW/cm^2^ irradiance.

### Microtensile bond strength test

After 24 h, the restored teeth were sectioned into resin–dentin sticks (1 mm^2^ of cross‐sectional area) and tested for microtensile stress in the universal testing machine DL2000 (EMIC‐Instron). Half of the sticks were tested immediately, and the other half was aged in PBS for 1 year before microtensile bond strength (µTBS) test [12, 13]. The µTBS values obtained from sticks of the same bonded tooth were averaged. The average bond strength of each tooth was used as a unit for statistical analysis. The µTBS data were statistically analyzed by two‐way ANOVA (factors Treatment and Age) and Tukey test with α = 5% after passing normality test (*p* = 0.71). Fractured specimens were analyzed by stereomicroscopy (40× magnification) and failures were classified as adhesive, cohesive in dentin, cohesive in composite or mixed (partial adhesive and cohesive fracture).

### Hydroxyproline assay

Measurement of hydroxyproline release was determined using a colorimetry assay kit (Sigma‐Aldrich), following the protocol described by Rifane et al. [13]. Briefly, six (*n* = 6) supernatants of storage PBS solutions collected after one year of aging, which contained standardized number of sticks (1 mm^2^ cross‐sectional area) from same bonded tooth were re‐suspended in 10 mL deionized water and tested following the manufacturer's instructions. Absorbance values were surveyed in UV–vis spectrophotometer (560 nm, Beckman Coulter DU‐800), standard curves of hydroxyproline (2, 5, 10, 15, and 25 mg/mL) were used as control and the solubilized collagen was expressed as mg of hydroxyproline per milliliter. The hydroxyproline data were statistically analyzed by one‐way ANOVA and Tukey's test (*p* < 0.05), after asserting normal data distribution (*p* = 0.815).

### Nanoleakage evaluation

One resin–dentin stick per tooth (*n* = 6, collected after one year of aging) was processed for silver nanoleakage evaluation as described by Tay et al. [14]. The silver‐impregnated specimens were analyzed in scanning electron microscopy (SEM) (JSM‐5600LV, JEOL) using the backscattered electrons mode.

### Confocal‐laser scanning microscopy

Further dentin specimens (*n* = 3) were prepared as previously described to evaluate the presence/activity of dentin MMPs at the bonded‐dentin interface through confocal‐laser scanning microscopy (CLSM). The number of specimens was chosen to obtain sufficient diversity of substrate, and not for statistical purposes as it is a qualitative analysis. A dye‐quenched MMP fluorophore based on gelatin was prepared by means of a fluorescein‐isothiocyanate (FITC) hypersaturated gelatin. Five milligrams of FITC were dissolved in 2 mL of 0.1 M sodium carbonate/bicarbonate buffer solution (pH 9.0, Sigma‐Aldrich). This reactant was added dropwise to a 1 mg/mL gelatin solution in the dark and incubated at room temperature for 2 h. Reacted FITC–gelatin conjugate was separated from unbound FITC using a G‐25 M Sephadex column [15].

The fluorescein/gelatin ratio was confirmed from absorbance readings at 495 and 280 nm in UV–vis spectroscopy. This MMP‐binding confocal dye [15] was dissolved (0.3 wt%) in distilled water and applied for 60 s after the pretreatments and before the bonding agent application. The adhesive was doped with 0.1 wt% of rhodamine‐B (Sigma‐Aldrich) for better visualization during confocal imaging at the interfaces [16]. The specimens were cut into 1‐mm thick resin–dentin slabs. Interfaces were analyzed through CLSM (LM710 Confocal, Carl Zeiss) equipped with a 63×/1.4NA oil immersion lens using a 488‐nm argon‐krypton ions laser illumination. The z‐stack scans were compiled into single projections. Each resin–dentin interface was entirely characterized, and images representing the MMP‐presence observed along the bonded interfaces were captured.

## RESULTS

### Synthesis of NMR‐MMPs

The synthesis of the metalloproteinase magnetic system to remove MMPs (NMR‐MMPS) proposed in the present study was successfully finalized with a final yield of ∼80%. Figure 1D,E shows the FTIR spectra of the final product and any intermediary product, demonstrating the presence of BB94 attached to the final magnetic nanoparticles.

### Microtensile bond strength

**FIGURE 2:**
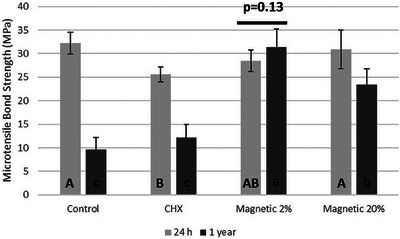
Outcomes of microtensile bond strength test. The bar above columns depicts no difference between 24 h and 1‐year aging. All further groups (Control, CHX, and Magnetic 20%) demonstrated bond strength reduction after aging. CHX means chlorhexidine. Different capital letters indicate significant difference among groups in 24 h period whereas different lowercase letters indicate statistically different bond strength among groups in 1‐year period (*p* < 0.05).

The overall microtensile outcomes are presented in Figure 2. The baseline outcomes (24 h) showed no significant difference between control, MAG‐2 and MAG‐20. Conversely, CHX treatment resulted in lower initial bond strength in comparison to the control group (*p* = 0.006). After 1‐year aging, control, CHX, and MAG‐20 groups demonstrated a significant bond strength reduction (*p* < 0.001), with the greatest drop in the control group. Contrariwise, MAG‐2 group presented a stable bond strength (*p* = 0.13) after prolonged aging. Failures were most in mixed mode in all groups, except for the specimens in the control group, which failed mainly in adhesive debonding after 1‐year aging.

### Hydroxyproline release

**FIGURE 3:**
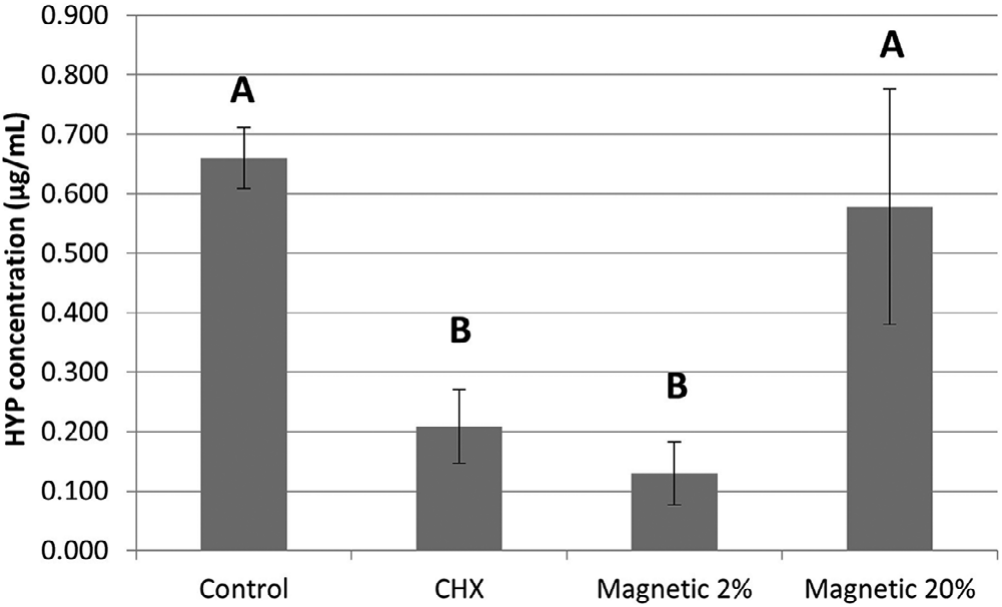
Amount of hydroxyproline (HYP) (µg/mL) released from the resin–dentin specimens in each group. The amount of HYP released from specimens was significantly different among the groups Control and MAG‐20 compared to chlorhexidine (CHX) and MAG‐2. Different letters indicate significant difference (*p* < 0.05).

The hydroxyproline release results are shown in Figure 3. Control and MAG‐20 presented the highest levels of collagen degradation, with no difference in terms of hydroxyproline release between these two groups (*p* = 0.802). However, both CHX and MAG‐2 treatment resulted in significantly lower hydroxyproline release than seen for the control treatment (*p* = 0.005 and *p* = 0.002, respectively). Overall, CHX reduced the collagen degradation by 68.3% compared to control, while MAG‐2 attained an 80.3% reduction.

### SEM‐nanoleakage

**FIGURE 4:**
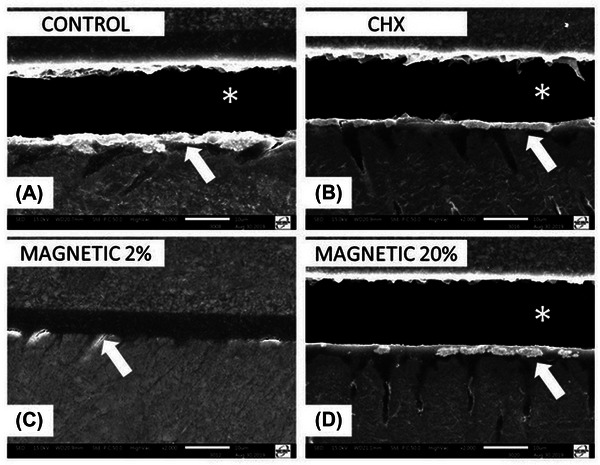
Scanning electron microscopy (SEM) micrographs of interfacial characteristics and nanoleakage after 1‐year aging. The asterisks represent hybrid layer failures and arrows highlight silver infiltration (nanoleakage). Control (A), chlorhexidine (CHX) (B), and Magnetic 20% (D) showed fractures in the hybrid layer after 1 year, while only Magnetic 2% (C) showed integrity of the surface. Control (A) showed striking silver uptake. CHX (B) and Magnetic 20% (D) showed similar areas of silver uptake. Magnetic 2% (C) showed very little nanoleakage after aging along all interfaces.

The most representative SEM images of resin–dentin interfaces are presented in Figure 4. All micrographs were obtained after 1 year of aging and except for the interfaces created with MAG‐2 treatment they all showed gaps along the interfaces due to degradation of the hybrid layer (asterisks in Figure 4A,B,D). Indeed, the MAG‐2 treatment preserved the resin–dentin bonding interface, which showed only very few silver deposits (Figure 4C).

### Presence/activity of MMPs in the interfaces

**FIGURE 5:**
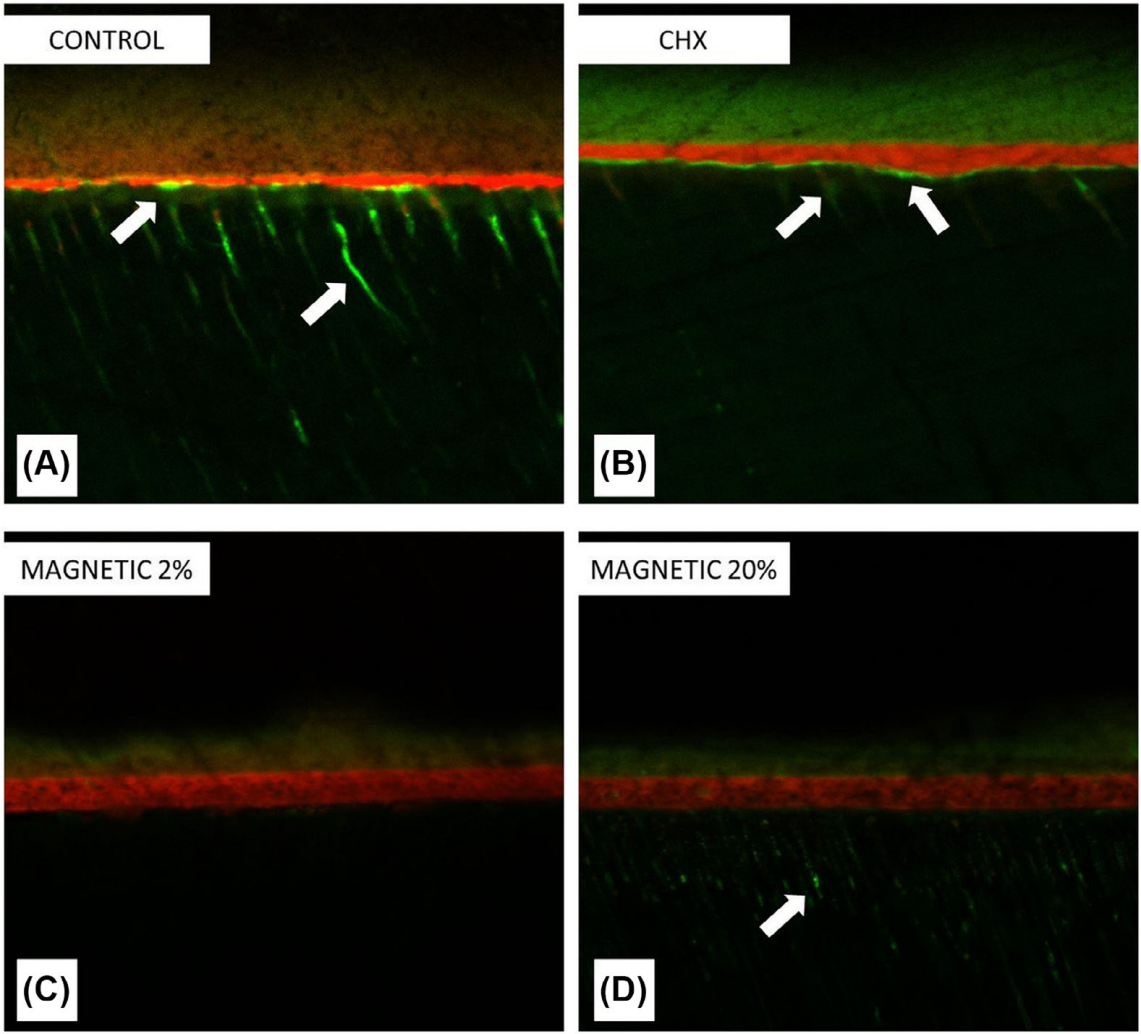
In situ zymography by confocal microscopy representing the presence and activity of dentinal MMPs. The arrows are highlighting the areas of matrix metalloproteinase (MMP) presence and activity as marked by MMP‐fluorescein fluorophore. All groups except Magnetic 2% depicted MMP presence and activity in hybrid layer for chlorhexidine (CHX) specimens (B), inside dentinal tubules for Magnetic 20% (D), or in both areas for Control (A). No presence of MMPs was detected along the interfaces of MAG‐2 specimens (C).

Representative CLSM images of each group are depicted in Figure 5. The presence of dentinal MMP, along with their collagen degradation activity were most evident in the control group, while only a slight MMPs presence/activity was observed in groups CHX (Figure 5B) and MAG‐20 (Figure 5D). Conversely, no detection of MMPs was observed in the specimens of group MAG‐2 (Figure 5C).

## DISCUSSION

According to the results obtained in this study, the first hypothesis was accepted, as the new nanoparticles for magnetic removal of MMPs (NMR‐MMPs) were able to remove MMPs from dentin matrix, especially at the lower concentration (2%) of NMR‐MMPs. Conversely, the second hypothesis that NMR‐MMPs increase dentin bonding longevity needs only partial acceptance because only the dentin treated with the gel containing 20% NMR‐MMPs presented a significant decrease in bond strength.

Batismastat (BB94) is a strong and well‐known MMP‐inhibitor [17], mainly used in biochemistry [18]. Even so, very few studies have investigated BB94 and its ability to promptly bind to dentin MMPs [19, 20]. The present study demonstrated by FTIR a strong ability of BB94 to attach onto the surface of Fe_3_O_4_ nanoparticles (Figure 1E). Indeed, the vibration bands in the region between 1250 and 1750 cm^‒1^ were only observed in Fe_3_O_4_@BPEI@BB94. Further evidence for the anchoring of BB94 to the nanoparticles can be found in the increase in relative intensity of the bands in the region between 2700 and 3000 cm^‒1^ (Figure 1D), which are attributed to the stretching of the CH2 group (νCH2) from BB94. Moreover, the vibrational mode at 1625 cm^‒1^ can be attributed to the stretching of carbonyl (νC  =  O) of amides that are generated by the anchoring between BB94 and BPEI molecules, and to the amide groups present in BB94 molecules (Figure 1E). The band at 1527 cm^‒1^ can be attributed to the secondary amides in BB94 and those formed during the reaction between BPEI and BB94 [10]. Notably, the FTIR spectra did not reveal additional bands indicative of aromatic iodination or other oxidative by‐products, suggesting that under the mild conditions used the coupling reaction was the predominant pathway, consistent with the protocol of Krishnamurthy et al [11]. The latter is due to the coupling between δN‒H and stretching of the C–N bond (νC‒N), a strong and stable bond that may ensure the proper application of the final product without releasing BB94. Additionally, the band at 1460 cm^‒1^ can be assigned to the stretching of the C = C bonds (νC = C) present in aromatic and thiophene rings of BB94 (Figure 1E). To the best of our knowledge, the anchoring of hydroxamic acids on amine‐coated magnetic nanoparticles has not been reported in the literature, and it is the key factor to synthesize the present magnetic nanoparticles able to attach to MMPs.

Among the substances employed to inhibit dentin MMPs, CHX is currently the main method used daily in clinical practice. Indeed, CHX has shown to exert great inhibition on MMPs [21, 22], and reduction of degradation of unprotected dentin collagen fibrils within the hybrid layer [1]. Several investigations confirmed the ability of CHX to extend the durability of resin–dentin bonds when used as primer/pretreatment [23, 24], in adhesives [25, 26], or in phosphoric acid gel [22]. However, further studies [27, 28] showed no significant improvements in long‐term adhesion after CHX application. Sadek et al. [29] showed that, after 18 months of incubation, the bond strength of CHX‐treated specimens was no longer stable. Despite the optimal initial enzymatic inhibition effect of CHX, water‐soluble CHX can leach out from dentin due to CHX substantivity and competing cations present in dentinal fluid [30, 31].

A meta‐regression analysis has indicated that the addition of CHX should be carefully analyzed before implementing new protocols for clinical adhesive procedures, as the association between the concentration of CHX and bond strength is not linear, and many other factors may affect bond strength [9]. More recently, a review concerning the clinical performance of bonded resin composite restorations created in combination with CHX primer application concluded that CHX attains no effect on final longevity of restorative fillings [32]. The current study showed that CHX induced partial inhibition of MMPs (Figure 5) and jeopardized dentin µTBS at 24 h. Furthermore, lack of stability was found in terms of dentin bonding after 1‐year aging (Figure 2). This could be explained by the vigorous water rinsing of all pretreatments, which might have reduced the active concentration of CHX. However, there is a contrast between loss of bond strength and hydroxyproline release, which may be related to acetone‐based adhesive used, which may further dilute CHX, reducing enzymatic inhibition, and the hydrolysis of resin components from resin–dentin specimens in the hydroxyproline assay.

Regarding treatment with NMR‐MMPs, both treatments (2 and 20 wt%) caused no alteration on initial µTBS. This may result from complete removal of nanoparticles by magnet approximation. A major concern in using the magnetic nanoparticles and magnetic motion is the possible aggressive alteration/cleavage of fibrils when the particles move across the demineralized collagen mesh, because of the enzymatic structures of MMPs when attached to the collagen fibril structure [33]. Indeed, this would yield consequent alteration of the substrate, particularly in high concentrations (i.e., 20%). We hypothesize that the high amount of nanoparticles in 20% NMR‐MMPs group may have infiltrated most demineralized dentin collagen fibrils, and with magnet approximation, the abrupt movement of all nanoparticles towards the same direction may have promoted the breakdown of some fibrils. Therefore, these partially fractured fibrils would be more prone to degradation causing a drop in µTBS. This might explain the lower results obtained for µTBS and hydroxyproline release with 20% NMR‐MMPS, which were similar to control group.

Conversely, a low concentration (2%) would seem able to remove the MMPs (Figure 5) without jeopardizing the structure of collagen fibrils and thereby favoring a stable dentin bonding and low total collagen degradation. After 1 year of aging, some collagen hydrolysis is expected, particularly within the areas of unprotected collagen fibrils in hybrid layers created when using simplified etch‐and‐rinse adhesives [34]. Such a slow degradation process can explain the results of hydroxyproline release, as little collagen degradation was detected with MMP inhibition by CHX or MMP removal by the treatment with NMR‐MMPs at 2% concentration, whereas high collagen breakdown was seen in the control group.

One concern related to the treatment with NMR‐MMPs is the possible negative antioxidant effect of BB94 on adhesive polymerization, which may cause reduction of bond strength. Indeed, the stable bond strength (in the case of 2% concentration) attained after removal of magnetic nanoparticles from dentin supports the idea that BB94 was also removed, along with MMPs. Furthermore, concerning some residual BB94 remaining within the demineralized dentin, a previous investigation [19] showed that adhesives containing BB94 demonstrated MMP inhibition in zymography, but with loss of bond strength after aging. Therefore, it is possible to state that the use of NMR‐MMPs does manage to remove the MMPs as demonstrated by confocal microscopy analysis (Figure 5) and confirmed by other experiments. In the oral environment, enzymatic activity may also derive from saliva, gingival crevicular fluid and potential release by pulp cells [8], which may later compromise longevity of composite restorations. Clearly, further studies are needed to confirm the ability/impact of removing MMPs with such magnetic treatment in clinical scenario.

The success of the synthesis of NMR‐MMPs represents a new strategy to overcome the issue of rapid enzymatic degradation of resin‐sparse demineralized dentin collagen. MMPs are not the only enzymes involved in this process. Cysteine cathepsins are a further class of endogenous proteases that may be activated during demineralization of sound and carious dentin [35]; however, the percentage of protonation (enzymatic properties) indicate that MMPs play a major role in enzymatic activity [8]. The outcomes of Tezvergil‐Mutluay et al. [36] demonstrated that collagen degradation promoted by MMPs is remarkably higher (67‐fold) than that attained with cathepsins, thus demonstrating that overall proteolysis in neutral and mildly acidic environments is accomplished mainly by MMPs. Nevertheless, future studies could assess similar magnetic nanoparticles for removal of cathepsins and their impact on dentin bonding and degradation. Additional future studies might focus on the action of NMR‐MMPs without using the external magnet and without creating magnetic motion, to assess the impact of solely external magnetic motion. A limitation of the present study is that the hydroxyproline assay is highly sensitive and could be affected by resin hydrolysis and pH changes during the aging period, Moreover, no direct test on extracted nanoparticles was performed to confirm the presence of MMPs. Clearly, there is further need for clinical trials to confirm whether the intervention can extend the longevity of resin composite restorations.

In conclusion, the nanoparticles proposed for magnetic removal of metalloproteinases may have an effective ability to remove MMPs from demineralized dentin matrix, thereby promoting stable dentin bond and diminishing collagen degradation.

## AUTHOR CONTRIBUTIONS

**Conceptualization**: Walter Zenobi, Davino Machado Andrade Neto, Karen Evellin Moura Cordeiro, Pierre Basilio Almeida Fechine, Diego Lomonaco, Garrit Koller, and Victor Pinheiro Feitosa; **Formal analysis**: Walter Zenobi, Salvatore Sauro, Davino Machado Andrade Neto, Karen Evellin Moura Cordeiro, Pierre Basilio Almeida Fechine, Diego Lomonaco, and Victor Pinheiro Feitosa; **Investigation**: Walter Zenobi, Davino Machado Andrade Neto, Karen Evellin Moura Cordeiro, Francisco Avelino, Yu Fu Chou, and Thiago Soares Porto; **Methodology**: Walter Zenobi, Davino Machado Andrade Neto, Karen Evellin Moura Cordeiro, Garrit Koller, and Victor Pinheiro Feitosa; **Writing—original draft**: Walter Zenobi, Davino Machado Andrade Neto, and Victor Pinheiro Feitosa; **Writing—review and editing**: Salvatore Sauro, Pierre Basilio Almeida Fechine, Diego Lomonaco, Thiago Soares Porto, and Victor Pinheiro Feitosa.

## CONFLICT OF INTEREST STATEMENT

The authors declare no conflicts of interest.

## FUNDING INFORMATION

This study was supported in part by the grant PID2020–120346GB‐I00 funded by AEI/10.13039/501100011033 “Ministerio de Ciencia, Innovación y Universidades” of Spain, and by Brazilian Ministry of Education CAPES grant AUXPE 23038.006958/2014‐96. Additionally, this work was partially funded by Fundação Cearense de Apoio ao Desenvolvimento Científico e Tecnológico (FUNCAP) (grant no. 07874997/2023).
